# Numerical simulation based performance enhancement approach for an inorganic BaZrS_3_/CuO heterojunction solar cell

**DOI:** 10.1038/s41598-024-57636-4

**Published:** 2024-03-31

**Authors:** Ahmed A. El-Naggar, Lotfy A. Lotfy, A. A. Felfela, Walid Ismail, Mahmoud Abdelfatah, Swellam W. Sharshir, Abdelhamid El-Shaer

**Affiliations:** 1 Physics Department, Faculty of Science, Kafrelsheikh University, Kafrelsheikh, 33516 Egypt; 2 Nano Science and Technology Program, Faculty of Science, Kafrelsheikh University, Kafrelsheikh, 33516 Egypt; 3 Mechanical Engineering Department, Faculty of Engineering, Kafrelsheikh University, Kafrelsheikh, 33516 Egypt

**Keywords:** Inorganic p–n heterojunction, CuO, BaZrS_3_, Solar cell simulator capacitance software (SCAPS-1D), Thickness, Bandgap, Carrier concentration, Materials science, Nanoscience and technology, Physics

## Abstract

One of the main components of the worldwide transition to sustainable energy is solar cells, usually referred to as photovoltaics. By converting sunlight into power, they lessen their reliance on fossil fuels and the release of greenhouse gases. Because solar cells are decentralized, distributed energy systems may be developed, which increases the efficiency of the cells. Chalcogenide perovskites have drawn interest due to their potential in solar energy conversion since they provide distinctive optoelectronic characteristics and stability. But high temperatures and lengthy reaction periods make it difficult to synthesise and process them. Therefore, we present the inaugural numerical simulation using SCAPS-1D for emerging inorganic BaZrS_3_/CuO heterojunction solar cells. This study delves into the behaviour of diverse parameters in photovoltaic devices, encompassing efficiency (η) values, short-circuit current density (J_sc_), fill factor (FF), and open-circuit voltage (V_oc_). Additionally, we thoroughly examine the impact of window and absorber layer thickness, carrier concentration, and bandgap on the fundamental characteristics of solar cells. Our findings showcase the attainment of the highest efficiency (η) values, reaching 27.3% for our modelled devices, accompanied by J_sc_ values of 40.5 mA/cm^2^, V_oc_ value of 0.79 V, and FF value of 85.2. The efficiency (η) values are chiefly influenced by the combined effects of V_oc_, J_sc_, and FF values. This optimal efficiency was achieved with CuO thickness, band gap, and carrier concentration set at 5 µm, 1.05 eV, and above 10^19^ cm^−3^, respectively. In comparison, the optimal parameters for BaZrS_3_ include a thickness of 1 µm, a carrier concentration below 10^20^ cm^−3^, and a band gap less than 1.6 eV. Therefore, in the near future, the present simulation will simultaneously provide up an entirely novel field for the less defective perovskite solar cell.

## Introduction

Governments across the world are relying more and more on renewable energy as a critical technology to handle energy-related environmental issues, such as lowering CO_2_ emissions^1^. Numerous OECD nations, most notably the US and Europe (where state policies and the EU Renewables Directive are prominently shown), have robust plans and objectives in place^2^. Renewable energy is also clearly supported by policy in developing countries such as China, India, and Brazil. The major focus is on the 'new' renewables: solar photovoltaics (PV), wind, and modern biomass^3^. While some of these technologies have made great strides and led to cost savings and rapid market expansion, others still need further research before they can be applied in a commercial setting^4,5^.

Solar photovoltaic (PV) energy systems were first utilised in space, but they are now widely used to generate electricity wherever it is needed^6^. Photovoltaic energy production is one of the most efficient and promising forms of renewable energy generation. Because PV technology has a little environmental effect, it is growing in popularity as a power generation method. Right now, hydropower and wind power are the two most widely used renewable energy sources in the world, with solar energy technology coming in third^7–9^. Additionally, the production of electricity using solar photovoltaic (PV) energy sources results in low carbon emissions, which reduces their impact on global warming^10^. Furthermore, power generated from fossil fuels emits between 400 and 1000 g of CO_2_ equivalent per kilowatt-hour (g CO_2_ eq/kWh), while silicon-based solar panels result in zero CO_2_ emissions^11^. Due to its clean, efficient, environmentally friendly, and reliable nature, solar energy through photovoltaic technology has a great deal of promise for supplying the world's energy needs in the future. PV panels that can produce sustainable energy on a global scale are in high demand due to the considerable increase in popularity of solar PV power in recent decades. Estimates indicate that by the end of the century, PV-generated electricity will surpass other global energy sources^12^.

Currently, the most efficient way to use solar radiation to generate energy is to employ multi-junction solar cells made of III–V compound semiconductors. Previous study has achieved efficiencies of up to 39% when exposed to concentrated sunshine^13^. These solar cells were initially designed to power satellites in space, but they are currently being used in conjunction with photovoltaic concentrator systems to investigate the market for terrestrial energy^14^. This generates an enormous potential market for compound semiconductor materials because of the vast regions required to catch adequate solar energy^15^. Concentrator systems based on III–V solar cells have demonstrated environmental friendliness and may be crucial to the future generation of sustainable energy^16^.

Modern society may be thought of having been built on semiconductors. Among other things, they are widely used in solar cells, power electronics, computer circuits, optical sensors, solid-state lasers, and light emitting diodes (LEDs). With a total of four levels of cation and anion coordination, covalent materials make up the vast majority of conventional semiconductors. However, because they compete with conventional semiconductors for photovoltaics in a way never seen before, organic–inorganic halide perovskites have garnered a lot of attention in the last 10 years^17–24^. These ionic materials have stronger coordination, which increases the Coulomb attraction between cations and anions. Strong ionicity is hypothesised to mitigate the risk of non-radiative carrier recombination resulting from deep level anti-site defects. Compared to ordinary semiconductors, halide perovskites have extraordinarily long carrier lifetimes (around 1 µs) and extremely low carrier concentrations (10^13^ cm^−3^)^25^. The efficiency of converting power in halide perovskite solar cells has experienced a remarkable growth, rising from 3.8% in 2009 to over 25% in 2019 Chalcogenide perovskite, a unique kind of semiconductor with the general formula ABX_3_, was recently found. It is made up of the following parts: A = Ca, Sr, Pb, Ba, Zn, Mg, Cd, Sn, and B = Zr, Hf, Sn, Si, Ge, Pb, Ti, and X = Se, S. They have a lower ionic concentration than oxides or halides, but they nevertheless have a higher ion concentration than regular semiconductors. These perovskite types have not received much attention despite being synthesised more than 50 years ago^26–32^, leading to a scarcity of knowledge on their physical attributes^33–35^. Following the screening of theory work, the chalcogenide materials ABX_3_ situation changed^36–38^. Numerous compounds with straight band gaps, high absorption rates, and favourable carrier mobility were found in the studies, suggesting that they may find use in optoelectronics. Numerous chalcogenide perovskites, including CaZrS_3_, SrZrS_3_, BaZrS_3_, SrHfS_3_ and SrTiS_3_, have been successfully synthesised. According to theoretical expectations, the chalcogenide perovskite BaZrS_3_, with an orthorhombic structure and a Pnma space group, crystallises with unit-cell parameters of a = 7.04, b = 9.98, and c = 7.05 and exhibits substantial light absorption^39,40^. Crucially, it was discovered that the material showed resistance to pressure, oxidation, and moisture^41^.

Several ABX_3_ chalcogenide perovskites with appropriate band gaps and absorption characteristics have been discovered for use in photovoltaics. Lately, researchers have been interested to the chalcogenide perovskite compound BaZrS_3_^42,43^. This compound is recognised as a novel class of photovoltaic semiconductors, whose gap value influences panel efficiency. BaZrS_3_, the prototype chalcogenide perovskite, with a linear band gap of 1.8 eV and significant near edge absorption^44^. Due to their remarkable chemical stability and absorption of visible light, chalcogenide perovskites (CPs), namely Barium Zirconium Sulphide (BaZrS_3_), have garnered considerable attention as a competitive alternative to hybrid halide perovskites in optoelectronics^45^. BaZrS_3_, a stable, non-toxic chalcogenide perovskite, is gaining attention for thin-film photovoltaic (PV) applications due to its superior stability and does not contain toxic elements compared to lead halide perovskites^46^.

One interesting oxide semiconductor is cupric oxide, often known as copper monoxide (CuO). Among other semiconducting oxides, It is a promising selective solar absorber due to its high solar absorbency and low thermal emittance^47,48^. It is suitable to solar cell applications due to its 1.2 eV optical band gap and its easy conversion to a p type material. In addition, it has a straight band gap, a high absorption coefficient, is readily synthesised, is inexpensive, abundant, and environmentally benign. Actually, the first CuO semiconductor humidity sensor was created in 1931 by Braver et al^5,49^. Due to its potential applications in lithium-ion batteries, flexible supercapacitors, optoelectronics, catalysis, fuel sensors, and as a superior absorbent to remove very harmful ions from ground water, this material has lately attracted a lot of attention.

The current study is working through the solar cell simulator capacitance program (SCAPS-1D) to study a highly efficient n–i–p CPs model solar cell^44,50^. SCAPS software is designed for the simulation of one-dimensional solar cells, specifically heterojunction and thin-film photovoltaic solar cells, enabling accurate modeling and simulation^51^.

Consequently, we are presenting SCAPS numerical simulations for novel inorganic BaZrS_3_/CuO heterojunction solar cell systems for the first time. In particular, we will investigate in detail how the window and absorber layers' bandgap, carrier concentration and thickness affect the fundamental characteristics of the solar cell. The results of our simulation will be a major first step towards determining the perfect conditions for high-efficiency solar device production.

## Structure of the apparatus and simulation approach

**Figure 1:**
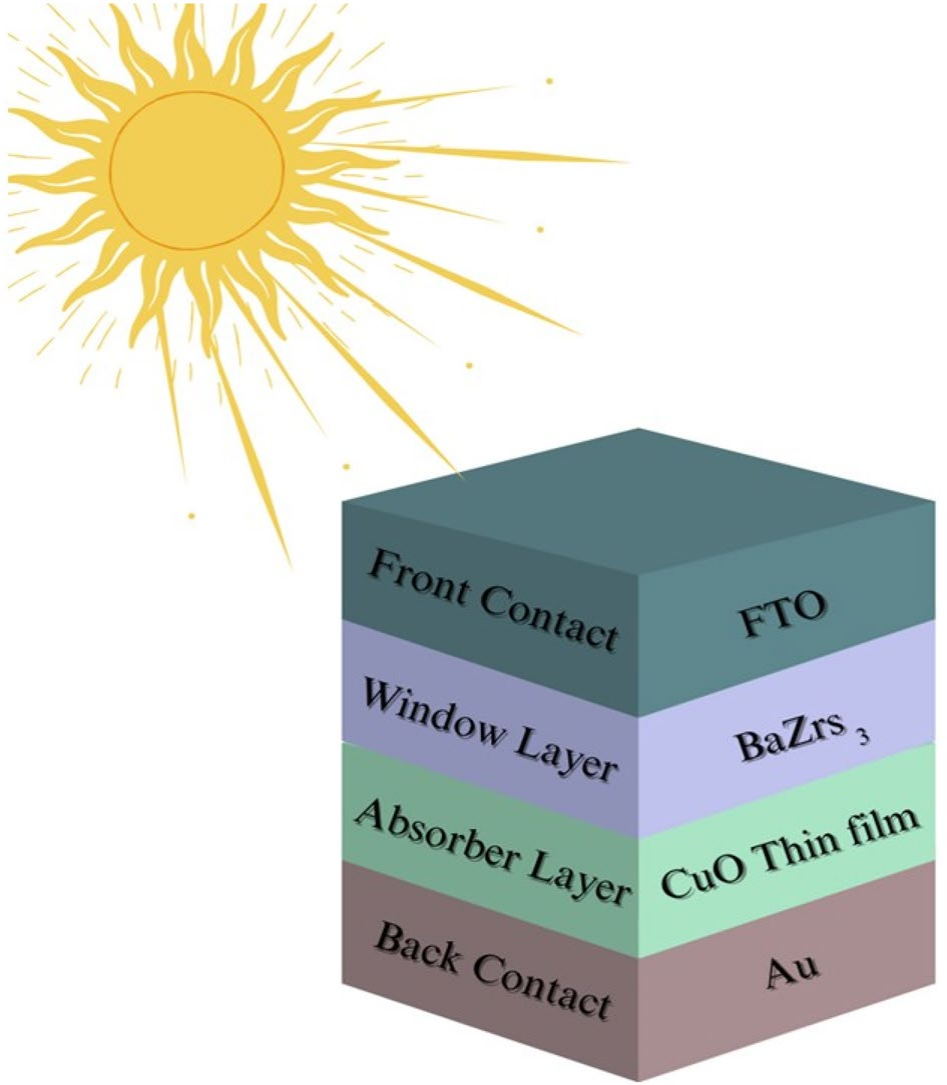
The structure of inorganic BsZrS_3_/CuO heterojunction solar cell.

In this study, CPs-based n–p planar perovskite device models have been constructed using solar cell simulator capacitance software structure (SCAPS-1D, version 3.3.07). In the manufacture of our inorganic p–n heterojunction solar cells, we often use an n-BaZrS_3_ thin film as the window layer and a p-CuO thin film as the absorber layer in order to solve the poisson and continuity equations. The device's front and back metal contacts were made of transparent conductive oxide (FTO) and gold (Au). A schematic diagram of the heterojunction device structure used in the simulation is shown in Fig. 1.

### Parameters used in the simulation

This research's main objective is to use the Solar Cell Capacitance Simulator Structures (SCAPS-1D) programme to simulate and analyse our inorganic solar cells. This programme is an advanced and useful numerical modelling tool designed to help comprehend and make sense of how solar systems actually function^46,52–54^. SCAPS software is a common choice for studying perovskite solar cells (PSCs) since it has advantages over alternatives such as Silvaco Atlas and GVPDM.SCAPS is free and open-source, offering a user-friendly interface and comprehensive manual, compared to the steep learning curve of Silvaco Atlas and GVPDM. SCAPS is flexible and customizable, allowing users to modify input parameters, material properties, and device structures according to their needs. It is fast and accurate, providing reliable results in a short time, unlike Silvaco Atlas and GVPDM, which are slow and complex, requiring more computational resources and time^55–57^. The construction and manufacturing of a solar cell device involve careful consideration of various parameters. These factors encompass the band gap, thickness, electron affinity, State effective densities in the valence band (V_B_) and conduction band (C_B_), dielectric permittivity, electron and hole mobilities, as well as shallow acceptor and donor densities (referred to as doping concentration and denoted as N_A_ and N_D_, respectively), and defect density N_t_. Exploring different values for these input variables is essential to identify optimal conditions for achieving enhanced performance in solar cell devices. Additionally, it is assumed that parameters like electron and hole thermal velocities remain consistent across all layers, each equal to 10^7^ cm/s. A detailed list of these parameters is provided in Table 1^46,58,59^, as well as the contact parameters used in the simulation are listed in Table 2^60,61^.

**Table 1:** Primary input parameters employed in the SCAPS-1D simulation for each material^46,58,59^.

Parameter of the material	n-BaZrS_3_	p-CuO
Thickness (nm)	500	100
Dielectric permittivity	9.6	18.1
Bandgap (eV)	1.76	1.2
Electron affinity (eV)	4.10	4.07
Effective density of states of valence band maximum (cm^−3^)	1.8 × 10^19^	5.5 × 10^20^
Effective density of states of conduction band minimum (cm^−3^)	2.2 × 10^18^	3 × 10^19^
Acceptor concentration (cm^−3^)	0	10^16^
Donor concentration (cm^−3^)	Wide range	0
Mobility of hole (cm^2^/(V s))	5.58	20
Mobility of electron (cm^2^/(V s))	11.3	200
Electron thermal velocity (cm/s)	10^7^	10^7^
Hole thermal velocity (cm/s)	10^7^	4.6 × 10^6^
Deffect density N_t_ (cm^−3^)	Wide range	1 × 10^5^

**Table 2:** The simulation's contact parameters^60,61^.

Contacts	Back metal contact properties (Au)	Front metal contact Properties (FTO)
Metal work function (eV)	4.98	4.07
Surface recombination velocity of electron (cm/s)	1.000 × 10^7^	1.000 × 10^7^
Surface recombination velocity of hole (cm/s)	1.000 × 10^7^	1.000 × 10^7^

However, the AM 1.5 light spectrum's intensity of light (100 mW/cm^2^) was utilised in all SCAPS-1D simulation calculations under standard testing conditions (STC). Using the following ratio, the continuity and poisson equations for electrons and holes were applied in SCAPS-1D^62,63^.

1 $$\frac{{d^{2} }}{{dx^{2} }}\Psi \left( x \right) = \frac{e}{{\varepsilon_{0} \varepsilon_{r} }} \left( {p\left( x \right) - {\text{n}}\left( {\text{x}} \right) + N_{D} - { }N_{A} + \rho_{p} - \rho_{n} { }} \right)$$

2 $$\frac{{dJ_{n} }}{dx} = {\text{G}} - {\text{R }}\quad {\text{and}}\quad \frac{{dJ_{p} }}{dx} = {\text{G}} - {\text{R}}$$

In this situation, the variables e (electron charge), Ψ (electrostatic potential), ε_0_ (vacuum permittivity), ε_r_ (relative permittivity), p (hole density), n (electron density), ρ_n_ (electron distribution), ρ_p_ (hole distribution), J_n_ (electron current densities), J_p_ (hole current densities), R (recombination rate), and G (generation rate) are denoted.

## Findings and analysis

### Analysis of the effects of CuO thickness, bandgap, and carrier concentration on the fundamental solar cell characteristics

Changing the absorber layer band gap, thickness, and carrier concentrations will have an initial influence on important photovoltaic variables that we are going to explore in this section. Specifically, we analyse the variations in open-circuit voltage (V_oc_), short-circuit current density (J_sc_), fill factor (FF), and cell efficiency (η). Throughout these investigations, we keep the window layer band gap, thickness, and carrier concentrations constant at values of 1.7 eV, 0.2 μm and 10^19^ cm^−3^, respectively.

**Figure 2:**
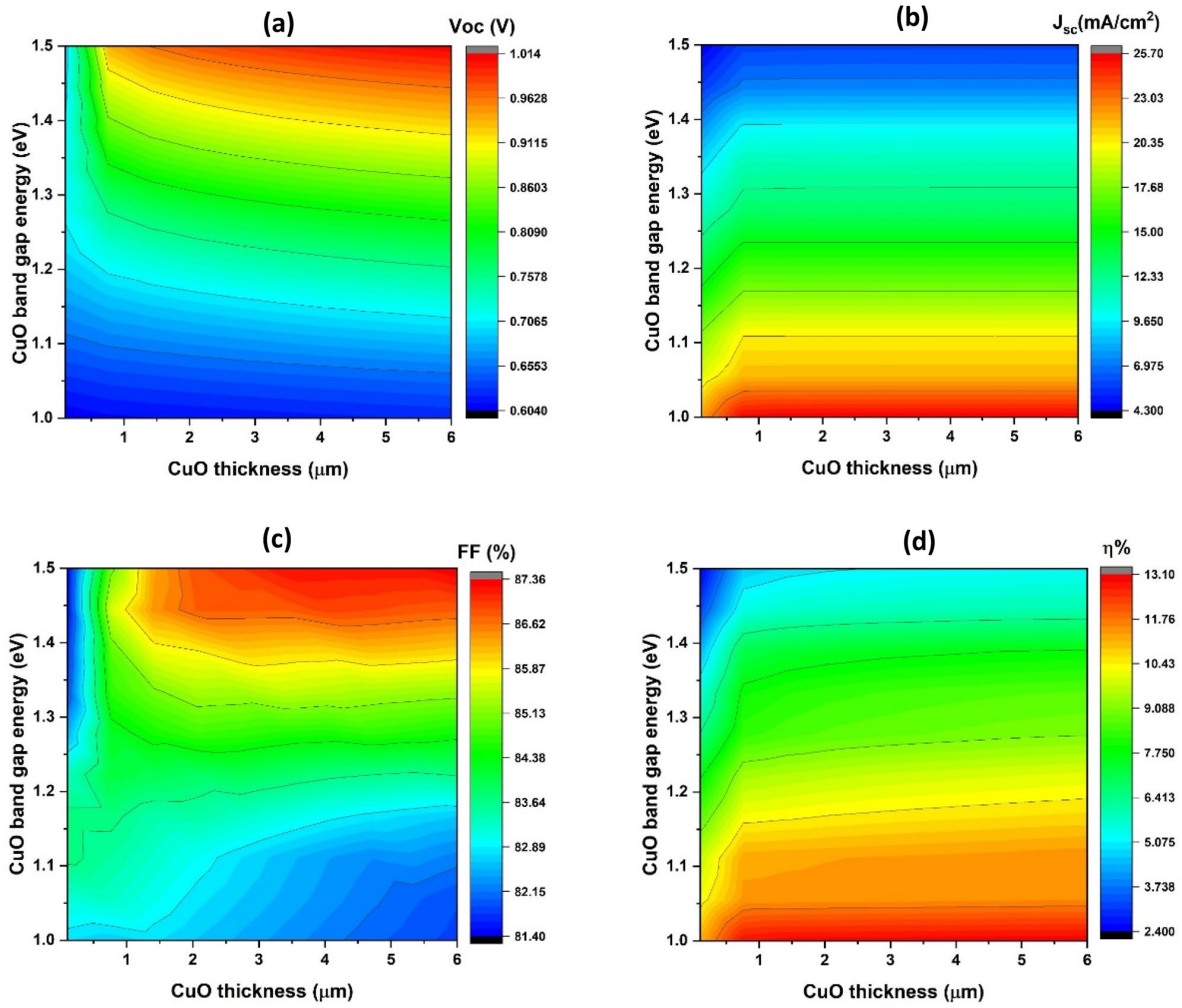
The basic characteristics of simulated solar cells with the absorber layer thickness (x-axis) and carrier concentration (y-axis).

As indicated in Fig. 2a, the open-circuit voltage (V_oc_) rises from roughly 0.6 V at a bandgap (E_g_) of 1 eV to approximately one V at an Eg of 1.5 eV. Meanwhile, the changes in V_oc_ with CuO thickness, spanning from 1 to 6 µm for any E_g_, do not exceed 0.1 V.

As depicted in Fig. 2b, the short-circuit current density (J_sc_) exhibits a decline, decreasing from approximately 25.7–4.3 mA/cm^2^ as the bandgap (E_g_) increases from 1 to 1.5 eV. Notably, the J_sc_ values remain relatively consistent across various CuO thicknesses, ranging from 0.5 to 6 µm.

In Fig. 2c, the fill factor (FF) values exhibit diverse trends. Firstly, there is a decrease from approximately 85.9% to about 81.4% as the bandgap (E_g_) increases from 1 to 1.5 eV, while increasing the thickness of CuO (thinner CuO thin films) from approximately 0.1–1.5 µm. Secondly, there is an increase from about 85.9% to approximately 87.3% with an increase in E_g_ from 1 to 1.5 eV. This is accompanied by a marginal 0.5% decrease in FF value as CuO thickness increases from roughly 1.5–6 µm (thicker CuO thin films).

In Fig. 2d, distinct zones with varying values are observed. A performance of approximately 12.2% is identified at a bandgap (E_g_) of about 1 eV, covering thicknesses ranging from about 0.5 to 6 µm. Across nearly every variation of E_g_ from 1 to 1.5 eV, an average value of approximately 11.2% is achieved, With the exception of the region with thickness between 2 and 6 µm and E_g_ between 1.3 and 1.45 eV, where a better performance of about 13.1% is obtained.

As per the provided equation, a rise in the band gap enhances the local collection efficiency of light absorption at the interface between the n-BaZrS_3_ thin film and the p-CuO thin film. This improvement leads to an augmented carrier generation rate, ultimately resulting in a notable increase in the V_oc_ value^64^ .

3 $$V_{oc} = \frac{{k_{B} {\text{T }}}}{{\text{q}}}\ln \left( {1 + \frac{{I_{ph} }}{{I_{0} }}} \right)$$

Furthermore, considering the bandgap values of BaZrS_3_ and CuO, an anticipated enhancement in the charge separation process is expected owing to a cliff-like band alignment^65^. The conduction band offset (ΔE_c_) value decreases as the bandgap energy increases because the valence band (E_v_) shifts downward and the conduction band (E_c_) shifts upward^65^. By making it easier for promoted electrons to travel from the absorber layer to the window layer interface, this decrease in ΔE_c_ promotes charge separation and raises V_oc_^65^. Additionally, an increase in band gap results in significant hole concentrations near the interface junction. As a result, the likelihood of recombination is increased due to heightened surface and interface recombination. This, in turn, accounts for the observed decrease in short-circuit current density (J_sc_)^65,66^. Moreover, the formation of charge carrier capture centers takes place due to the wavefunction crossover between vibrationally excited states of lower and higher-lying electronic states^66^.

Fill factor (FF) behavior is always affected by a number of parameters, but the series and shunt resistances are particularly important. The V_oc_ values also play a role, albeit to a lesser extent, as illustrated in the following equation^67^.

4 $${\text{FF}} = \frac{{{{\varvec{\upupsilon}}}_{oc} - {\text{ln}}\left( {{{\varvec{\upupsilon}}}_{oc} + 0.72} \right)}}{{{{\varvec{\upupsilon}}}_{oc} + 1}}$$

where υ_oc_ = qV_oc_/AkT

The behavior of η and values of V_oc_, J_sc_, and FF can be illustrated using the equation provided below^68^.

5 $${\upeta } = \frac{{FF V_{oc} J_{sc} }}{{ P_{in} }}$$

**Figure 3:**
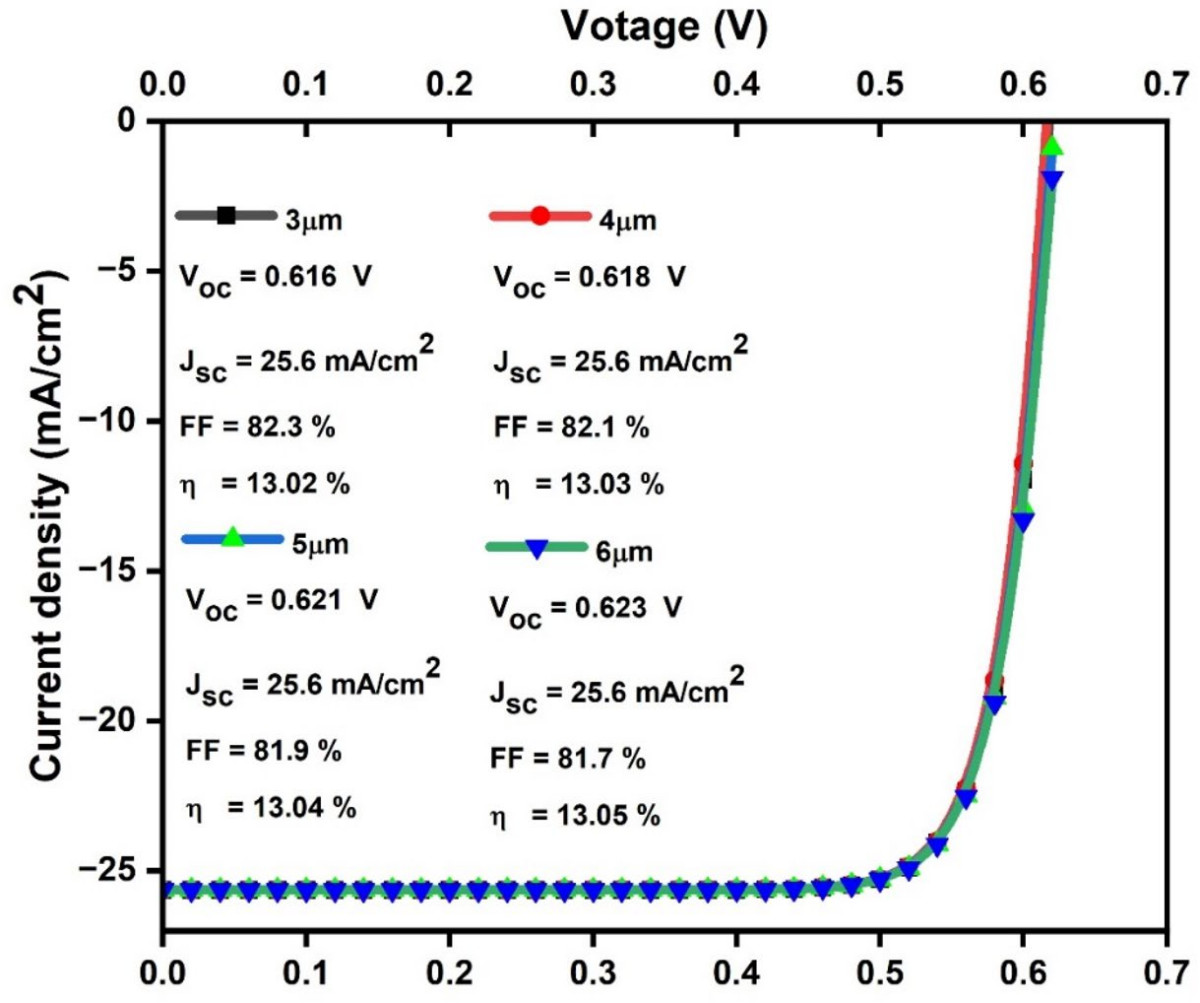
Modelled JV curves with basic characteristics of solar cells as a function of CuO thickness in the range of 3–6 µm.

Based on the preceding data, we have inferred that, for enhanced solar cell efficiency, it is advisable to maintain the CuO band gap between 3 and 6 µm in thickness and 1.05 eV in range. As a result, as shown in Fig. 3, we generated JV curves that represent the essential properties of solar cells as a function of CuO thickness, which ranges from 3 to 6 µm, while maintaining a constant band gap of 1.05 eV. It clearly indicates that V_oc_ ranges from 0.616 to 0.623 V, J_sc_ remains constant at 25.6 mA/cm^2^, FF varies between 81.7 and 82.3%, and η ranges from 13.02 to 13.05 each having a thickness that increases from 3 to 6 µm. Notably, the η values closely follow the trends observed in V_oc_ and FF values.

**Figure 4:**
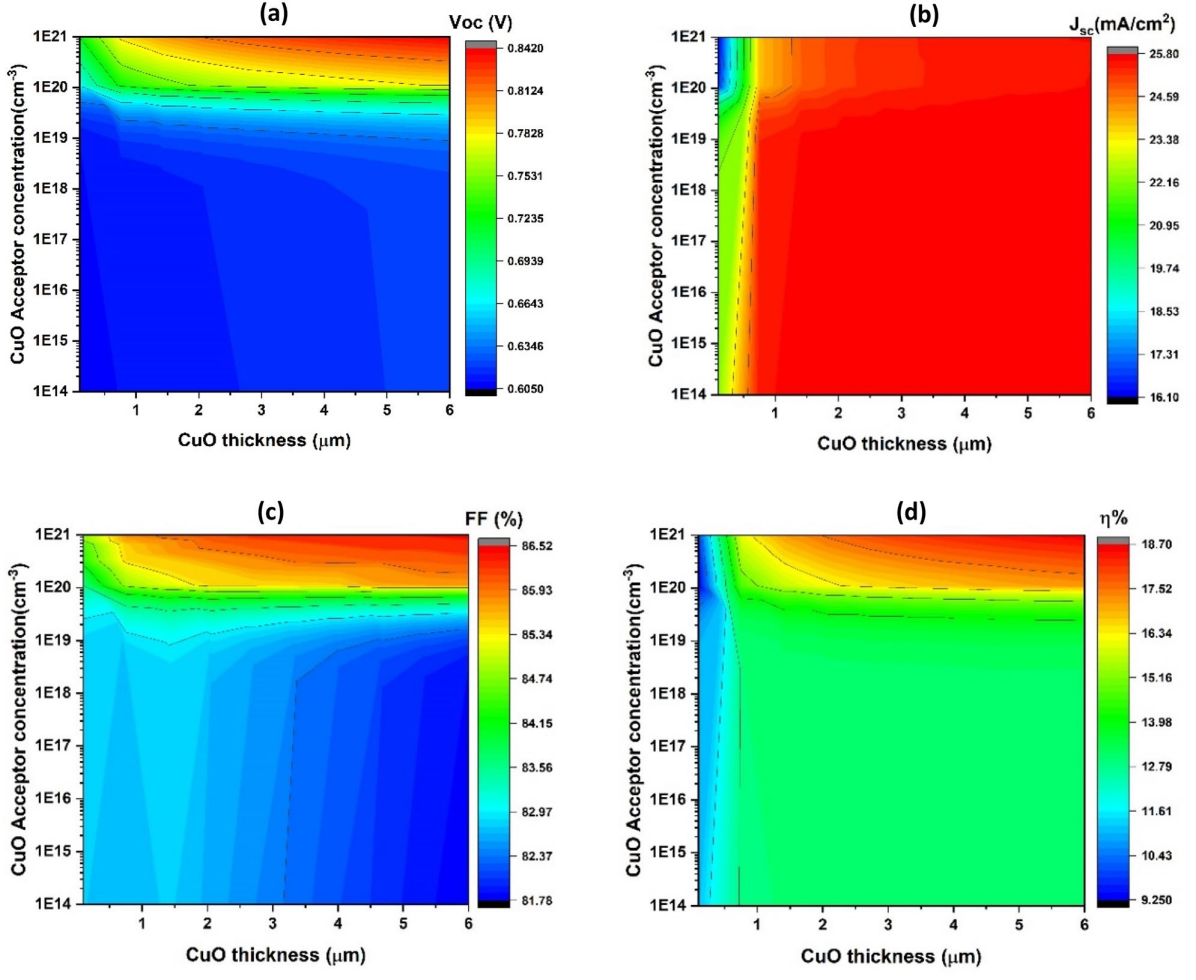
Fundamental parameters of simulated solar cells, including V_oc_ (**a**), J_sc_ (**b**), FF (**c**), and η (**d**), with a constant bandgap (E_g_) of 1.05 eV, as functions of the (absorber layer thickness on the x-axis) and (carrier concentration on the y-axis).

Figure 4 shows a contour plot with the obtained photovoltaic parameters V_oc_, J_sc_, FF, and η as functions of the carrier concentration from 10^14^–10^21^ cm^−3^ (y-axis) and CuO thin film thickness from 1 to 6 µm (x-axis).

As depicted in Fig. 4a, the open-circuit voltage (V_oc_) rises from approximately 0.6–0.84 V as the acceptor concentration increases from 10^14^ to 10^21^ cm^−3^. There are only minimal variations in V_oc_ values as CuO thickness increases.

According to Fig. 4b, the short-circuit current density (J_sc_) increases from approximately 23.3–25.8 mA/cm^2^ when the acceptor concentration remains constant, ranging from 10^17^ to 10^21^ cm^−3^. It maintains constancy at around 25.5 mA/cm^2^ for carrier concentrations below 10^20^ and for CuO thicknesses exceeding 0.6 µm. For CuO thicknesses less than 0.6 µm, the J_sc_ remains approximately 22.3 mA/cm^2^ for carrier concentrations exceeding 10^20^ cm^−3^, but the J_sc_ value varies with CuO thickness, ranging from 23.3 to 25.8 mA/cm^2^ for carrier concentrations below 10^20^ cm^−3^.

In Fig. 4c, it is observed that the fill factor (FF) demonstrates an increase with thickness, reaching approximately 84.1–86.5% within the thickness range of 1–6 µm. For a thickness greater than 1 µm and a carrier concentration of 10^21^ cm^−3^, the FF is notably higher at around 86.5%, indicating a positive correlation with thickness. This suggests that FF values mostly remain constant, hovering around 84.15%, for a carrier concentration of 10^20^ cm^−3^, and decrease with increasing thickness for carrier concentrations below 10^20^ cm^−3^.

Figure 4d illustrates that the values of η exhibit an increase from approximately 15.2% to about 18.7% with an increase in carrier concentration from 10^19^ to 10^21^ cm^3^ and as the thickness increases. The η value remains constant at 13.2% when the concentration of the carriers is less than 10^19^ cm^−3^. A thickness greater than 3 µm and a carrier concentration of 10^21^ cm^−3^ result in the higher η value of roughly 18.7%.

With the increase in carrier concentration, From the p-CuO thin film to the n-BaZrS_3_ thin film, there is increased electron diffusion. Consequently, the device's built-in voltage (V_bi_), primarily determined by the depletion width (W_d_), experiences amplification, leading to a significant rise in V_oc_. Additionally, the increase in photogenerated current (electron–hole pairs) (I_ph_) contributes to the reverse saturation current increasing and the recombination rate decreasing(I_o_)^69^. This phenomenon is reflected in Eq. (3), which elucidates the correlation between V_oc_, I_ph_, and I_o_, demonstrating how an increase in carrier concentration results in higher V_oc_ values. On the contrary, as the carrier concentration increases, there is an increase in the population of photogenerated charge carriers and a simultaneous reduction in both leakage current and recombination current^70^. This effect is particularly noticeable for CuO thicknesses exceeding 1.6 µm.

When the carrier concentration exceeds 10^19^ cm^−3^ and the absorber thickness is less than 1.6 µm, there is an increase in J_sc_ with CuO thickness. This rise in thickness can be attributed to the charge-collecting length of CuO, enhancing the junction's electron–hole pair collecting^71^. It indicates that as the thickness increases, the charge carriers are more effectively separated and stored. When the carrier concentration is above 10^19^ cm^−3^ and the CuO thickness surpasses 1.6 µm, the charge-collecting length is maximized. This leads to an increased probability of carrier collection, a reduction in recombination rate, and a decrease in leakage current, resulting in larger J_sc_ values^65^. As demonstrated in Eq. (5), the behavior of η can be understood as the combined effect of V_oc_, J_sc_, and FF, with η being a function of these parameters^68^.

**Figure 5:**
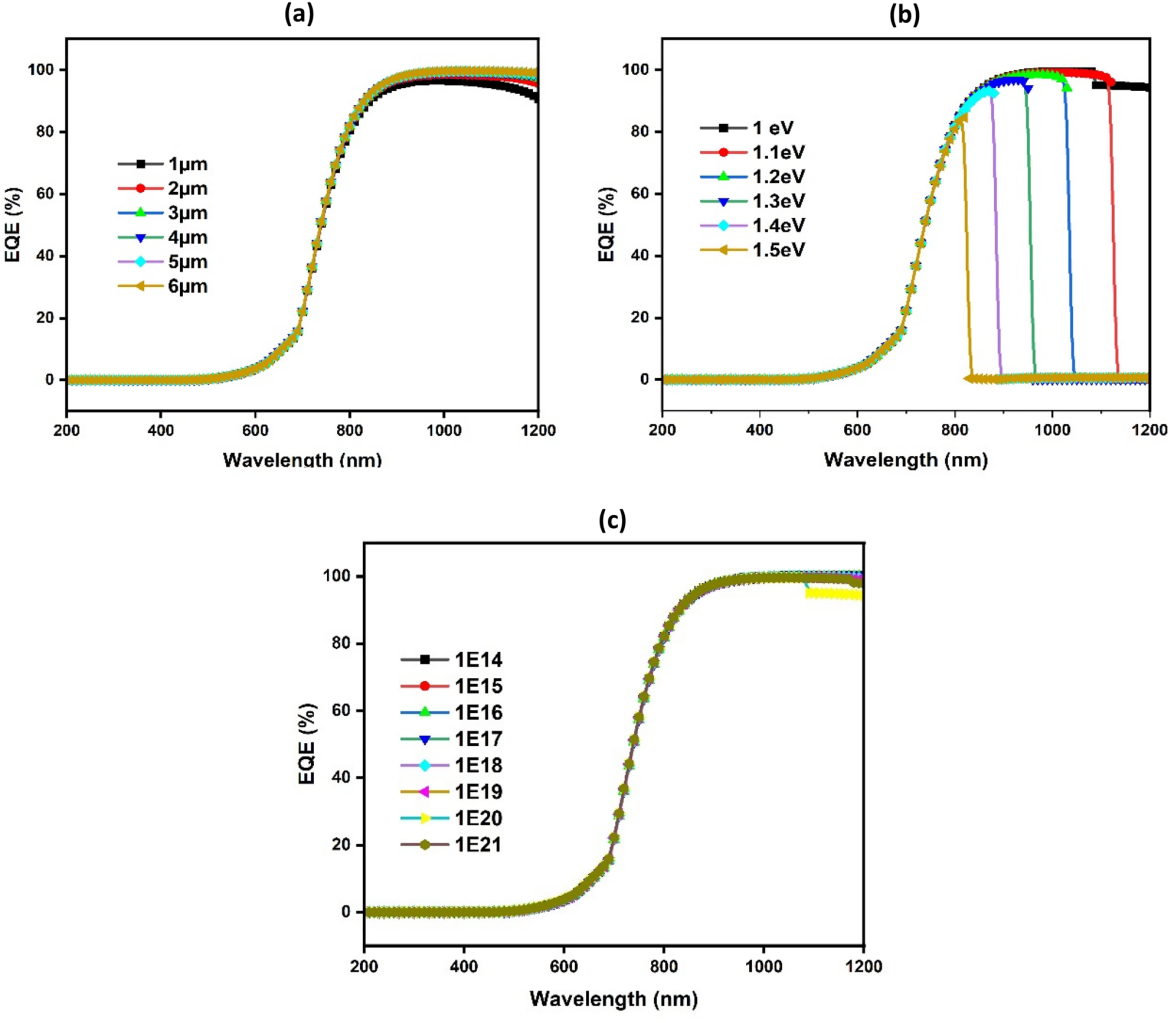
Simulated external quantum efficiency with CuO thickness (**a**), band gap (**b**), and carrier concentration (**c**), respectively.

In this scenario, the shape of EQE predominantly mirrors the shape of J_sc_, as acceptor concentration primarily influences photogenerated current. Consequently, It is determined what the external quantum efficiency (EQE) is in relation to the band gap, carrier concentration, and CuO thickness, as depicted in Fig. 5. With EQE at 0% at 200 nm and 100% at 800 nm, it is clear that all CuO thicknesses follow a comparable pattern. The greater EQE component's area under the curve drops from 1200 to 800 nm when the band gap raises from 1 to 1.5 eV, and it increases by raising the carrier concentration from 10^14^ to 10^21^ cm^−3^. In addition, there is an intrinsic absorption edge in the short-wavelength region of the spectrum. Variations in carrier concentration along with reflection off the Au back contact may cause these edges to shift in position.

Studies show that when high-energy photons are present, the photogenerated current rises and decreases with low-energy photons, suggesting varying rates of creation and recombination of charge carriers that impact J_sc_. These findings validate and suggest that η is primarily influenced by J_sc_. Based on the preceding calculations, it can be inferred that for BaZrS_3_/CuO heterojunction solar cell devices to achieve higher efficiencies, the optimal ranges for CuO, thickness, carrier concentration and band gap are approximately 5 µm, above 10^19^ cm^−3^ and 1.05 eV, respectively.

### Impact of bandgap, carrier concentration, and thickness of BaZrS_3_ on fundamental solar cell characteristics

Furthermore, we will explore the impact of variations in the thickness, bandgap, and carrier concentrations of the BaZrS_3_ thin film, serving as the window layer, on crucial features of photovoltaic devices. Meanwhile, the thickness of the CuO layer, bandgap, and carrier concentrations will be held constant at 5 µm, 1.05 eV, and 10^19^ cm^−3^, respectively.

**Figure 6:**
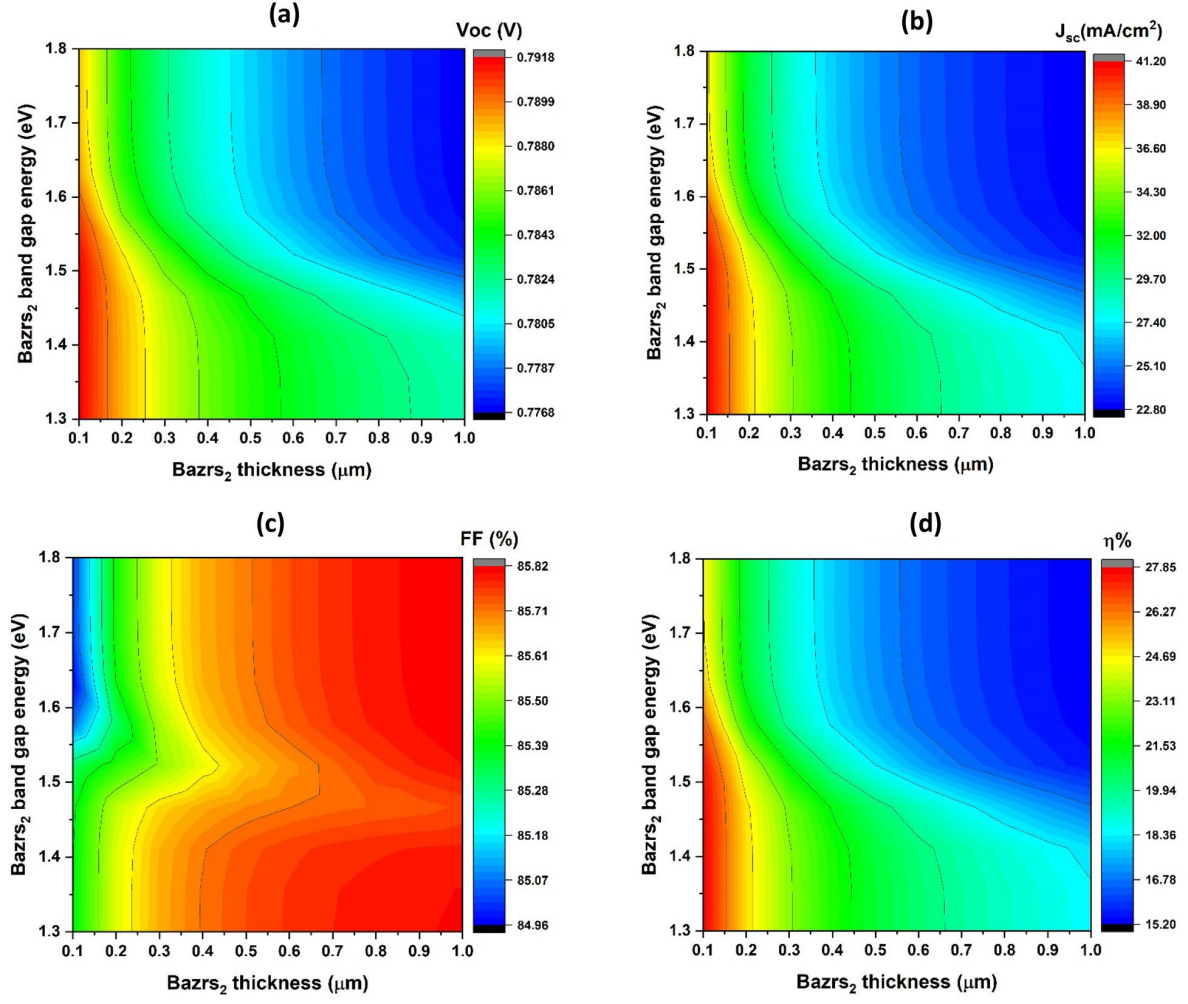
Critical parameters of simulated solar cells, showcasing open-circuit voltage (V_oc_) (**a**), short-circuit current density (J_sc_) (**b**), fill factor (FF) (**c**), and efficiency (η) (**d**) with respect to the band gap (y-axis) and window layer thickness (x-axis), maintaining a constant donor concentration of 10^19^ cm^−3^.

Figure 6 presents a contour map reflecting the simulated fundamental characteristics of a solar cell, illustrating how these properties change as the bandgap (which varies from 1.3 to 1.8 eV on the y-axis) and BaZrS_3_ thin film thickness (which ranges from 0.1 to 1 µm on the x-axis) change.

Figure 6a illustrates the variations in open-circuit voltage (V_oc_) at different points. Specifically, as thickness increases from 0.1 to 1 µm, it drops from roughly 0.79 V to roughly 0.77 V, with the maximum V_oc_ observed at approximately 0.1 µm thickness. Furthermore, for smaller band gaps ranging from 1.3 eV to around 1.6 eV, there is an improvement in the V_oc_ value at each bandgap value.

The J_sc_ values in Fig. 6b follow a similar pattern to the V_oc_, decreasing from around 41.2 mA/cm^2^ to approximately 22.8 mA/cm^2^ as thickness increases from 0.1 to 1 μm. The peak J_sc_ is observed at approximately 0.1 μm in thickness. Lower band gaps ranging from 1.3 eV to about 1.6 eV lead to an increase in the J_sc_ value for each corresponding bandgap value.

In Fig. 6c, the FF values exhibit multiple zones where the FF increases from 84.96 to 85.82% as the thickness increases from 0.1 to 1 μm. An average FF value of approximately 85.8% is achieved for nearly all variations of the E_g_ from 1.3 to 1.8 eV, except for the zone situated at the E_g_ from 1.4 to 1.5 eV and the thickness from 0.6 to 1 μm, which attains a higher performance of around 85.8%.

In Fig. 6d, the η values exhibit a similar trend to the V_oc_, where η decreases from around 27.85% to about 15.2% as the thickness increases from 0.1 to 1 µm. For band gaps less than 1.6 eV and thicknesses less than 0.1 µm, an average value of approximately 27% is observed.

The obtained results can be understood in the following manner: More light enters the interface junction when the band gap increases, leading to a higher rate of photogenerated charge carriers. This, in turn, facilitates the separation and collection of carriers, while concurrently reducing both recombination current and leakage current. These combined effects contribute to an elevation in the V_oc_ value, particularly for band gaps less than 1.6 eV, as explained in Eq. (3)^64^. Moreover, the reduction in the V_oc_ value is associated with an increase in thickness. This augmentation promotes the generation of electron–hole pairs across the junction by extending the charge collection length^71^. Additionally, bandgap alignment plays a crucial role in enhancing the V_oc_ value. Smaller band gaps in BaZrS_3_, especially those less than 1.6 eV, lead to increased confinement, resulting increasing the current due to increased carrier intra-band tunnelling at the interface voltage barrier^72^. A similar rationale can be applied to explain the behaviour of J_sc_ values.

Increasing the band gap of BaZrS_3_ leads to a reduction in both V_oc_ and J_sc_ values. This is attributed to an increase in the recombination process and leakage current, particularly noticeable as the thickness of BaZrS_3_ increases^65,66^. The mismatch between the band gaps of CuO and BaZrS_3_ in this scenario results in the creation of capture centres, promoting carrier recombination^66^.

As indicated in Eq. (4), the behaviour of the fill factor (FF) can be explained by its inverse relationship with V_oc_ values. The efficiency (η) values are predominantly influenced by the combined effects of V_oc_, J_sc_, and FF values, as outlined in Eq. (4)^68^.

Our findings suggest that maintaining the thickness of BaZrS_3_ between 0.1 and 0.2 µm, decreasing with thickness, and setting the band gap below 1.6 eV can enhance device efficiency.

**Figure 7:**
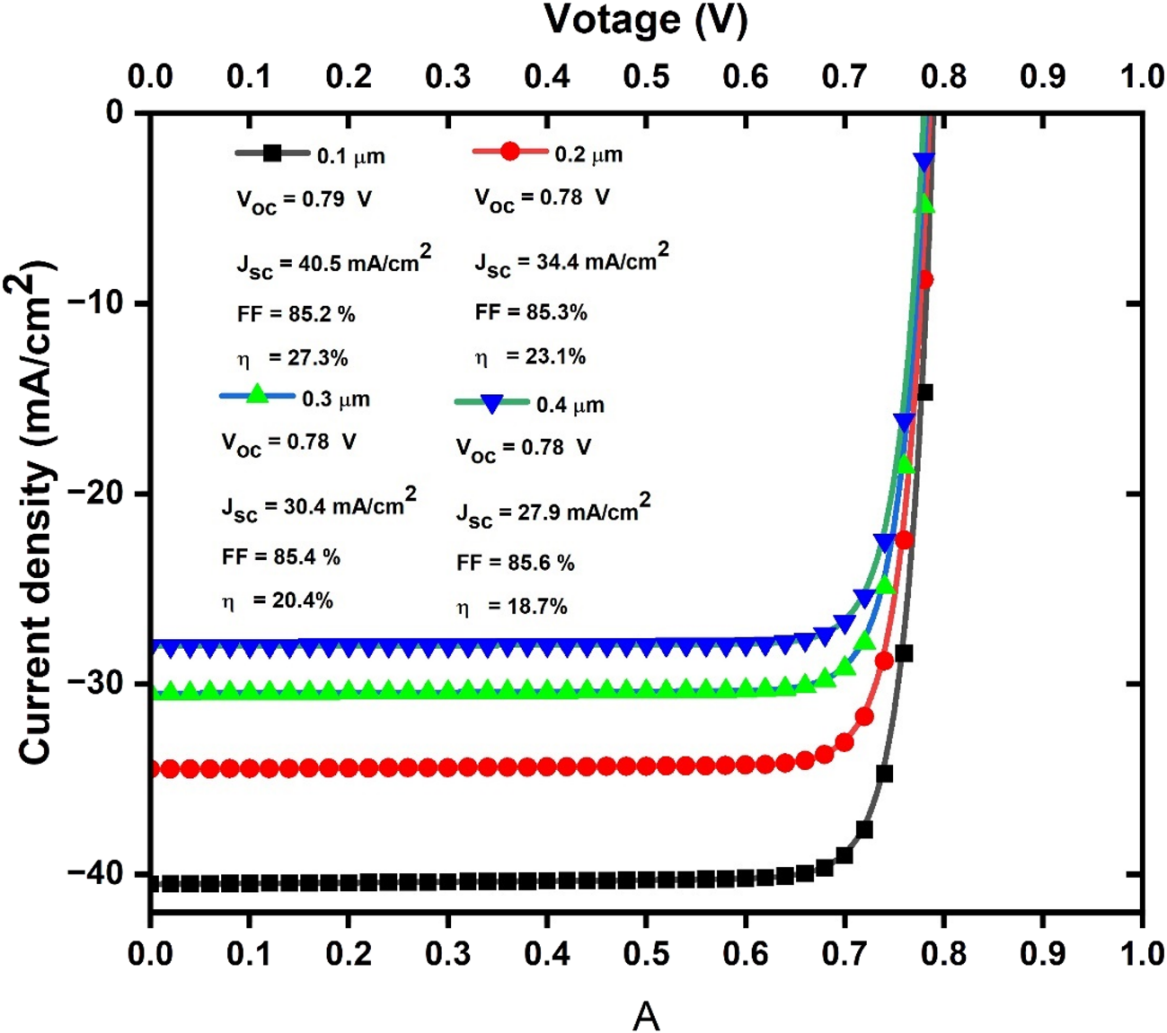
Current–voltage (JV) curves of solar cell devices with BaZrS_3_ thickness varied from 0.1 to 0.4 µm.

Under these conditions, as shown in Fig. 7, the thickness increases from 0.1 to 0.4 µm, the fill factor (FF) ranges from 85.2 to 85.6%, the efficiency (η) decreases from 27.3 to 18.7%, the open-circuit voltage (V_oc_) varies between 0.78 and 0.79 V, and the short-circuit current (J_sc_) decreases from 40.5 to 27.9 mA/cm^2^.

**Figure 8:**
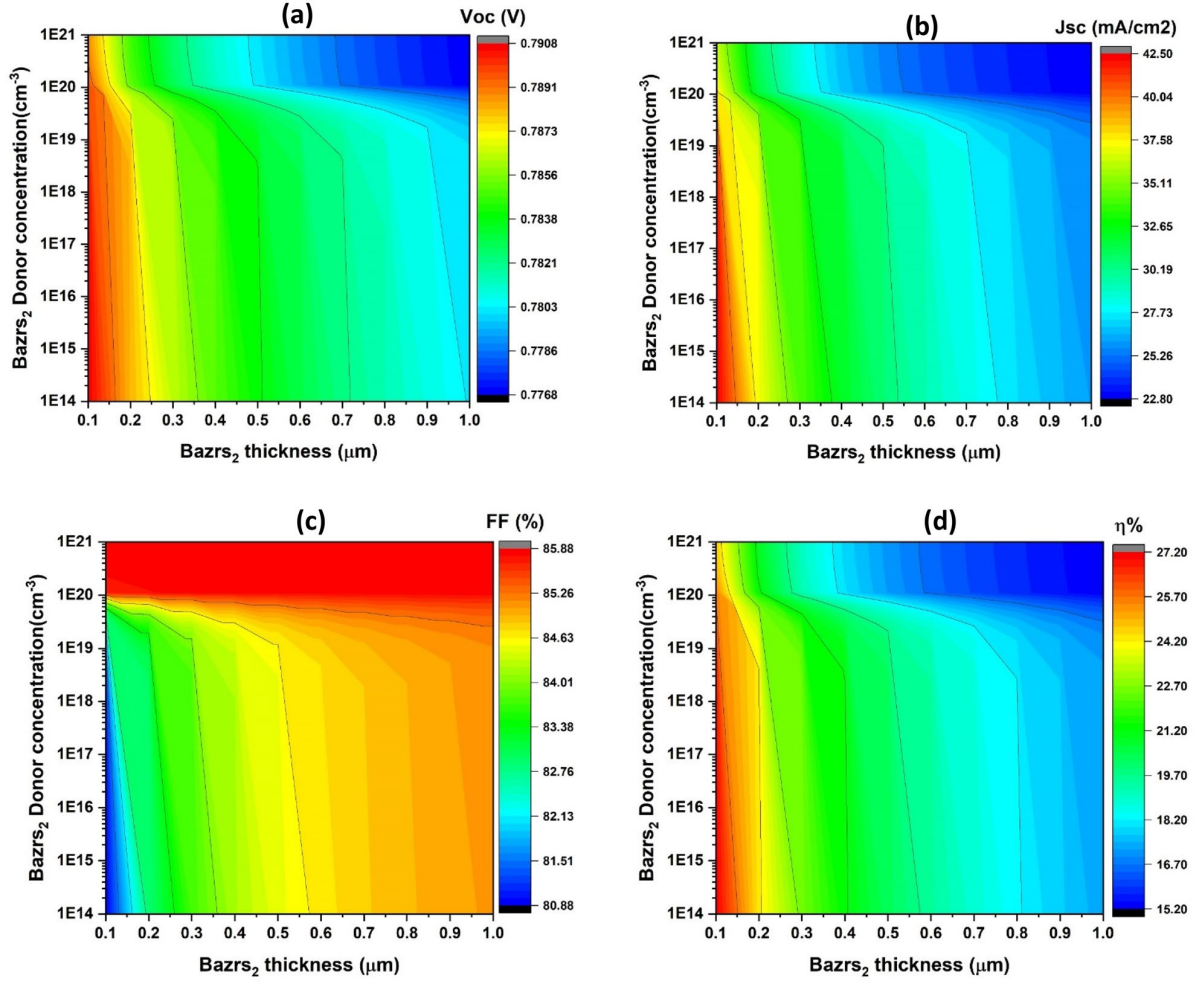
Critical parameters of simulated solar cells; open-circuit voltage (V_oc_) (**a**), short-circuit current density (J_sc_) (**b**), fill factor (FF) (**c**), and efficiency (η) (**d**) with respect to the band gap (y-axis) and window layer thickness (x-axis), maintaining a constant donor concentration of 10^19^ cm^−3^.

Figure 8 depicts a contour plot demonstrating variations in critical solar cell characteristics, where the carrier concentration ranges from 10^14^ to 10^21^ cm^−3^ on the y-axis and the BaZrS_3_ thin film thickness, which ranges from 0.1 to 1 μm, is the dependent variable.

The study of Fig. 8a demonstrates that the open-circuit voltage (V_oc_) varies across distinct regions. More precisely, it falls from roughly 0.79 V to roughly 0.77 V as thickness rises from 0.1 to 1 μm, while the highest V_oc_ reached at thickness approximately 0.1 μm. When carrier concentrations are less than 10^20^ cm^−3^, the V_oc_ value improves for each given carrier concentrations value.

Figure 8b indicates that the J_sc_ values follow the same pattern as the V_oc_, with J_sc_ decreasing from approximately 42.5 mA/cm^2^ to approximately 22.8 mA/cm^2^ as thickness increases from 0.1 to 1 μm and the highest J_sc_ attained at thickness of approximately 0.1 μm. The J_sc_ value improves for each individual carrier concentration value when the carrier concentration is less than 10^19^ cm^−3^. According to Fig. 8c the FF value remained constant at around 85.8% for carrier concentrations more than 10^20^ cm^−3^ with increasing BaZrS_3_ thickness. The FF value rises from 0.1 to 1 μm as the thickness increases when the carrier concentration is less than 10^20^ cm^−3^. In contrast, the FF reached its maximum value for levels of carrier concentration greater than 10^20^ cm^−3^. Figure 8d, demonstrate that the η values have the same form as the V_oc_, which decreases from about 27.2% to about 15.2% as the thickness increases from 0.1 to 1 μm, while the highest achieved at thickness around 0.1 μm. The value improves for every individual carrier concentration value in the event that the carrier concentration is below 10^19^ cm^−3^.

The data clearly show that increasing the donor concentrations of the BaZrS_3_ thin film decreases the V_oc_ value as the thickness increases, especially when the concentration is less than 10^19^ cm^−3^ for each thickness. This absence of influence could be due to the entire establishment of the depletion width (W_d_), which influences the charge carrier generation rate and minority carrier diffusion length. The flow of electrons in the p-CuO layer and holes in the n-BaZrS_3_ layer influences the photogenerated current significantly^65^. The full built-in voltage (V_bi_) and the depletion width (W_d_), primarily determined on the p-CuO thin film side, are directly associated with this current^64^. For each given carrier concentration below 10^20^ cm^−3^, the V_oc_ value drops as the BaZrS_3_ thickness increases. This is due to the longer lifetime and larger charge collection channel, which results in enhanced photogenerated current while concurrently decreasing leakage current and recombination rates as thin film thickness increases^71^.

These observations are corroborated by the fact that raising the carrier concentration above 10^20^ cm^−3^ results in a drop in J_sc_ value, even while the thickness increases. Furthermore, the decrease in J_sc_ is due to the window layer predominantly functioning as a conducting channel for electrons rather than contributing considerably to solar spectrum absorption^73^.

**Figure 9:**
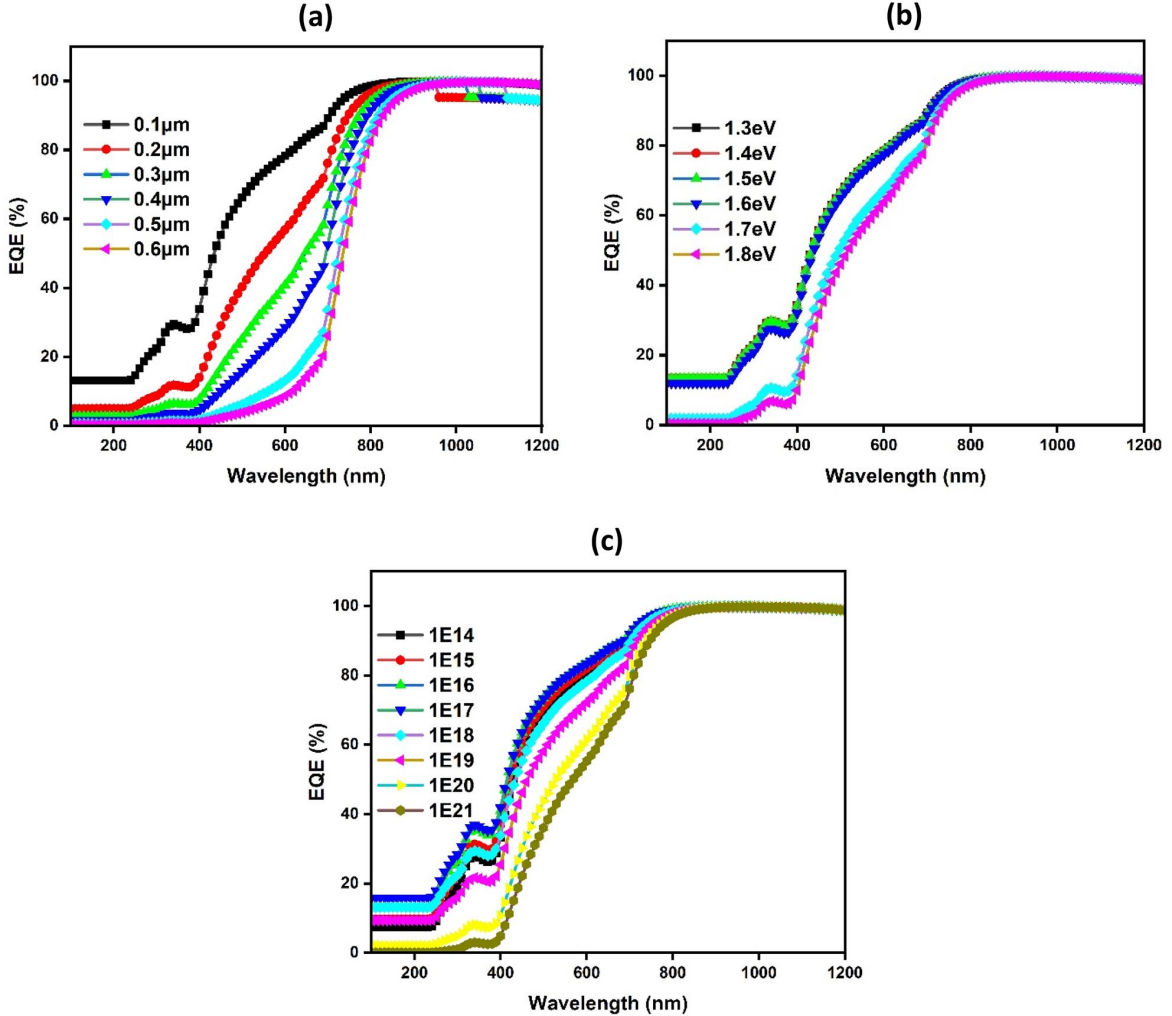
Simulated (EQE) as dependent variables of BaZrS_3_ thickness (**a**), Band gap (E_g_) (**b**), and carrier concentration (**c**).

The efficiency (η) values provide a complete view of performance through the combination of V_oc_, J_sc_, and FF, with its value predominantly following V_oc_ and FF trends. As shown in Fig. 9, photogenerated current is investigated by simulating EQE as a function of BaZrS_3_ band gap, carrier concentration and thickness.

It is clear that EQE drops from approximately 18% to nearly zero as thickness increases from 0.1 to 0.6 m, particularly at higher energy levels, and from approximately 18% to almost zero when energy levels increase from 1.3 to 1.8 eV, particularly at longer wavelengths. This decrease in EQE with band gap and thickness is caused by an increase in recombination rate, which lowers charge carrier collection and, in turn, lowers the produced current's separation^74^. It is important to note that, independent of carrier concentration, EQE has the same shape and value. The values of J_sc_ for the obtained BaZrS_3_ thin film band gap, carrier concentrations and thickness are consistent with and supported by the EQE results.

Additionally, our modelling outcomes indicate that the ideal values for CuO band gap, carrier concentration and thickness in inorganic BaZrS_3_/CuO heterojunction solar cell devices should be approximately 1.05 eV, above 10^19^ cm^−3^ and 5 µm, respectively. In comparison, the optimal parameters for BaZrS_3_ are a thickness of 1 µm, a carrier concentration of less than 10^20^ cm^−3^, and a band gap of less than 1.6 eV. With a V_oc_ value of 0.79 V, a J_sc_ value of 41.2 mA/cm^2^ and an FF value of 85.8%, the maximum efficiency was attained under these conditions.

Our results show that solar cell devices based on BaZrS_3_/CuO could compete with those based on In_2_S_3_. By contrast, Cu(In, Ga)Se_2_, CuIn(S,Se)_2,_ and Cu_2_ZnSnS_4_-based solar cells with In_2_S_3_ as the buffer layer (thickness: 50–125 nm), fluorine-doped tin oxide as the window layer, and gold (Au) as the back contact showed the best efficiency, ranging from 16.94 to 22.50%^75^ .Reyes et al. also created an n–i–p heterojunction perovskite solar cell with the following configuration: FTO/TiO_2_/MASnI_3_/Cu_2_ZnSnS_4_/Au. The best values were obtained with donner and acceptor densities of 10^16^ cm^−3^ and 10^14^ cm^−3^, respectively, and comprised a J_sc_ of 31.66 mA/cm^2^, a V_oc_ of 0.96 V, an FF of 67%, and an efficiency (η) of 20.28%^76^.

### Effect of operating temperature on JV curves and the fundamental specifications of the ideal BaZrS_3_/CuO solar cell

**Figure 10:**
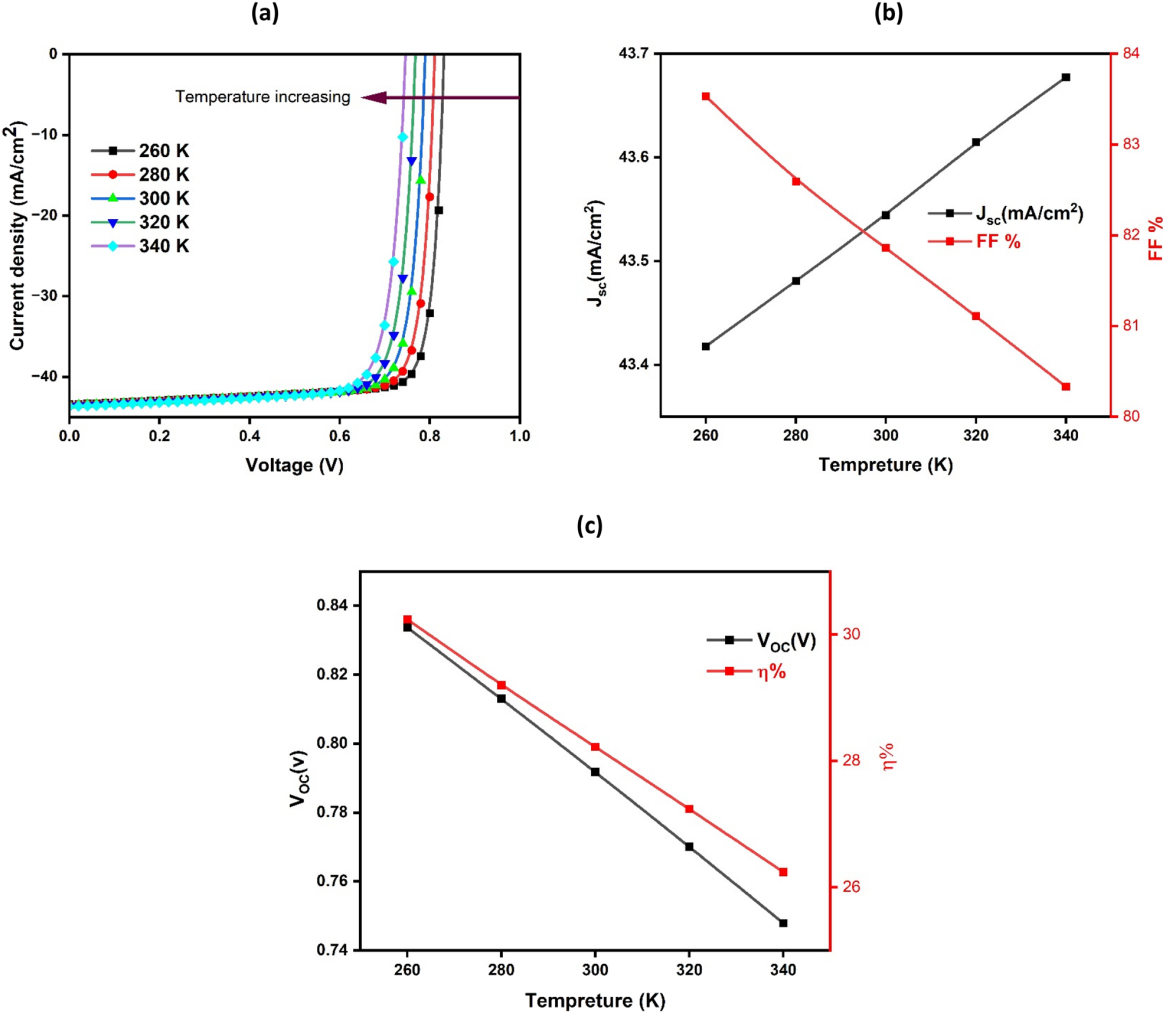
Simulated JV curves and crucial variables of the optimal BaZrS_3_/CuO solar cell, showcasing their variations with operational temperature, measured in Kelvin (K).

In this section, we examined the impact of operational temperature on the JV curves and key parameters of the most efficient BaZrS_3_/CuO solar cell, as illustrated in Fig. 10. It is evident that an increase in operational temperature leads to a reduction in both V_oc_ and J_sc_, resulting in a decrease in the overall efficiency (η). These values decrease from 0.834 to 0.747 V, 43.67 to 43.41 mA/cm^2^, and 30.2% to 26.2% as the temperature rises from 260 to 340 K, respectively.

The results shown can be clarified through Eq. (3), in which the reverse saturation current (I_o_) increases with increasing temperature, as does the chance of charge carrier recombination. As a result, V_oc_, J_sc_, and value all fall. It is worth mentioning, however, that the variation in η is relatively small, especially when compared to average global temperatures. Under typical temperature settings, the BaZrS_3_/CuO solar cell may still be a feasible option for making high-performance photovoltaic systems.

Table 3 highlights the simulated photovoltaic performance of various device structures, showcasing distinct voltage, current density, fill factor, and efficiency metrics. Notably, the work stands out with a device structure featuring FTO/BaZrS_3_/CuO/Au, exhibiting promising characteristics with a voltage at open circuit of 0.79 V, a short-circuit current density of 40.5 mA/cm^2^, a fill factor of 85.2%, and an efficiency of 27.03%.

**Table 3:** Comparison of the current simulation result with the predicted maximum PSG for photovoltaic performance.

Device structure	Voltage at Open Circuit V_OC_ (V)	Short-circuit current density J_SC_ (mA/cm^2^)	Fill factor FF (%)	Efficiency η (%)	References
FTO/MAPbl_3_/Au	0.823	17.53	82.67	12.35	^35^
ITO/ZnO/MAGeI_3_/Spiro- OMeTAD/Au	1.74	16.27	64.51	18.3	^54^
FTO/TiO_2_/BaZrS_3_/Cu_2_O/Au	1.16	12.24	87.13	12.42	^46^
FTO/TiO_2_/BaZrS_3_/Spiro-OMeTAD/Au	1.21	16.54	86.26	17.29	^77^
FTO/TiO_2_/BaZrS_3_/CuSbS_2_/W	1.00	22.57	73.7	17.13	^78^
TCO/Zrs_2_/CuO/Au	0.96	34.2	72.2	23.08	^58^
FTO/TiO_2_/Ba(Zr_0.87_,Ti_0.12_)S_3_/Cu_2_O/back contact	1.09	26.57	85.78	24.86	^79^
FTO/TiO_2_/BaZrSe_3_/Spiro-OMeTAD/Au	0.72	46.65	77.32	25.84	^80^
AZO/i-ZnO/CdS/Ba(Zr_0.95_,Ti0_.05_)S_3_/a-Si	1.26	27.06	88.47	30.06	^81^
FTO/ZrS_2_/BaZrS_3_/SnS/Pt	1.18	29.74	80.15	28.17	^82^
FTO/ BaZrS_3_/CuO/Au	0.79	40.5	85.2	27.03	This work

Comparing these results to prior studies, it's evident that different device architectures yield varying performance outcomes. For instance, while some configurations demonstrate higher V_OC_ or J_SC_ values, this structure exhibits a competitive combination of these parameters, alongside a notable FF and the efficiency. This suggests the potential of BaZrS_3_/CuO as an active layer in photovoltaic devices, particularly in achieving balanced performance across key metrics.

## Conclusions

Chalcogenide perovskites have emerged as promising materials with unique optoelectronic characteristics and enhanced stability, garnering increasing interest among researchers. However, challenges such as high-temperature requirements and lengthy reaction durations have constrained the synthesis and processing of these materials.

To address this, we present the initial numerical simulation using SCAPS-1D for novel inorganic BaZrS_3_/CuO heterojunction solar cells. Copper oxide has low thermal emittance and strong solar absorbency, making it a viable selective solar absorber among the various semiconducting oxides being studied. And, compared to lead halide perovskites, BaZrS_3_, a stable, non-toxic chalcogenide perovskite, is becoming increasingly common for thin-film photovoltaic applications because of its superior stability and absence of hazardous components.

This study comprehensively explores various parameters in photovoltaic devices, encompassing efficiency (η), short-circuit current density (J_sc_), fill factor (FF), and open-circuit voltage (V_oc_). We also thoroughly investigate the impact of window and absorber layer thickness, carrier concentration, and bandgap on the fundamental characteristics of solar cells. Our outcomes reveal that factors such as built-in voltage (V_bi_), depletion width (W_d_), charge carrier collecting length, photogenerated current, minority carrier lifetime, and recombination rate significantly influence solar cell performance.

Our results demonstrate the attainment of the highest efficiency (η) values, reaching 27.3% for our modeled devices, accompanied by J_sc_ values of 40.5 mA/cm^2^, V_oc_ value of 0.79 V, and FF value of 85.2. The Fill factor (FF) is influenced by series and shunt resistances and exhibits an inverse relationship with V_oc_ values. The efficiency (η) values are predominantly influenced by the combined effects of V_oc_, J_sc_, and FF values. This optimal efficiency was achieved with CuO thickness, band gap, and carrier concentration set at 5 µm, 1.05 eV, and above 10^19^ cm^−3^, respectively.

In comparison, the optimal parameters for BaZrS_3_ include a thickness of 1 µm, a carrier concentration below 10^20^ cm^−3^, and a band gap less than 1.6 eV. Our simulated results underscore the growing attention toward organic–inorganic halide perovskites in photovoltaics due to their potential for high efficiency, large-scale, low-cost fabrication, and reduced non-radiative carrier recombination. These perovskites exhibit stronger coordination, enhancing Coulomb attraction between cations and anions, contributing to their favourable properties.

### Future work

We can Investigate the effect of different metal contacts, such as Ag, Cu, Fe and C on the performance and stability of BaZrS_3_/CuO PSCs and exploring the potential of other chalcogenide perovskites, such as BaSnS_3_, BaTiS_3_, and BaHfS_3_, as absorber materials for PSCs.

## Data Availability

The datasets used and/or analyzed during the current study available from the corresponding author on reasonable request.
